# Surgical intervention for portal hypertension caused by oxaliplatin-based chemotherapy: a case report and a review of literature regarding radiological and/or surgical interventions for oxaliplatin-associated portal hypertension

**DOI:** 10.1007/s12328-020-01157-w

**Published:** 2020-06-26

**Authors:** Daisuke Morioka, Yusuke Izumisawa, Kazuya Yamaguchi, Kei Sato, Satoshi Komiyama, Kazuya Nakagawa, Manabu Kakizoe, Takashi Murakami, Yoshiki Sato

**Affiliations:** 1 Department of Surgery, Yokohama Ekisaikai Hospital, 1-2 Yamada-cho, Naka-ku, Yokohama, 231-0036 Japan; 2 Gastroenterology Unit, Gastroenterological Center, Yokohama City University, Yokohama, Japan; 3 Department of Surgery, Teikyo Chiba Medical Center, Chiba, Japan

**Keywords:** Oxaliplatin-based chemotherapy, Portal hypertension, Portosystemic shunt

## Abstract

A 63-year-old man showed massive ascites, massive pleural effusion, severe lower-extremity edema, and repeated esophageal variceal bleeding. Two-year previously, he received 13-courses of oxaliplatin-based chemotherapy (OBC) followed by associating liver partition and portal vein ligation for staged hepatectomy (ALPPS) for multiple colorectal cancer liver metastases but developed a solitary remaining liver metastasis and multiple lung metastases 2 months after the ALPPS, for which multiple regimens of chemotherapy were conducted. The symptoms were considered attributable to the OBC-associated portal-hypertension. Water-retention symptoms were mitigated by the use of tolvaptan but the variceal bleeding necessitated frequent endoscopic treatments and disallowed restarting antineoplastic treatment. Transjugular intrahepatic portosystemic shunt (TIPS) was considered undesirable because TIPS in this patient might have prohibited future repeat hepatectomy. Thus, the patient underwent splenectomy and surgical portosystemic shunting. Since then, the portal-hypertension symptoms were completely resolved. Thereafter, chemotherapy was able to be recommenced. Moreover, repeat hepatectomy was performed. A literature review demonstrated that radiological and/or surgical interventions for the OBC-associated portal-hypertension have been reported in 31 cases to date. However, this report is the first to show a case of successful treatment of the OBC-associated portal-hypertension with splenectomy and surgical portosystemic shunting, which allowed subsequent chemotherapy followed by repeat hepatectomy.

## Introduction

The oxaliplatin-based chemotherapy (OBC) has been currently the standard first-line treatment for unresectable colorectal cancer since 2000 when its efficacy was proven [1, 2]. Although the sinusoidal obstruction syndrome (SOS) has been reportedly observed in 20–50% of the patients receiving the OBC [2], the OBC-associated portal-hypertension due to SOS is considered relatively rare [3–15]. However, it can cause potentially life-threatening complications, such as massive ascites, pancytopenia, and gastrointestinal variceal bleeding [3–15]. To prevent and/or treat these complications, the radiological and/or surgical interventions are sometimes required, such as partial splenic embolization (PSE) [10], splenic arterial ligation [9], or transjugular intrahepatic portosystemic shunt (TIPS) [7].

Herein, this report is the first to show a case of the severe OBC-associated portal-hypertension successfully treated by surgery consisting of splenectomy and surgical portosystemic shunting, which allowed subsequent chemotherapy followed by repeat hepatectomy.

## Case report

**Fig. 1:**
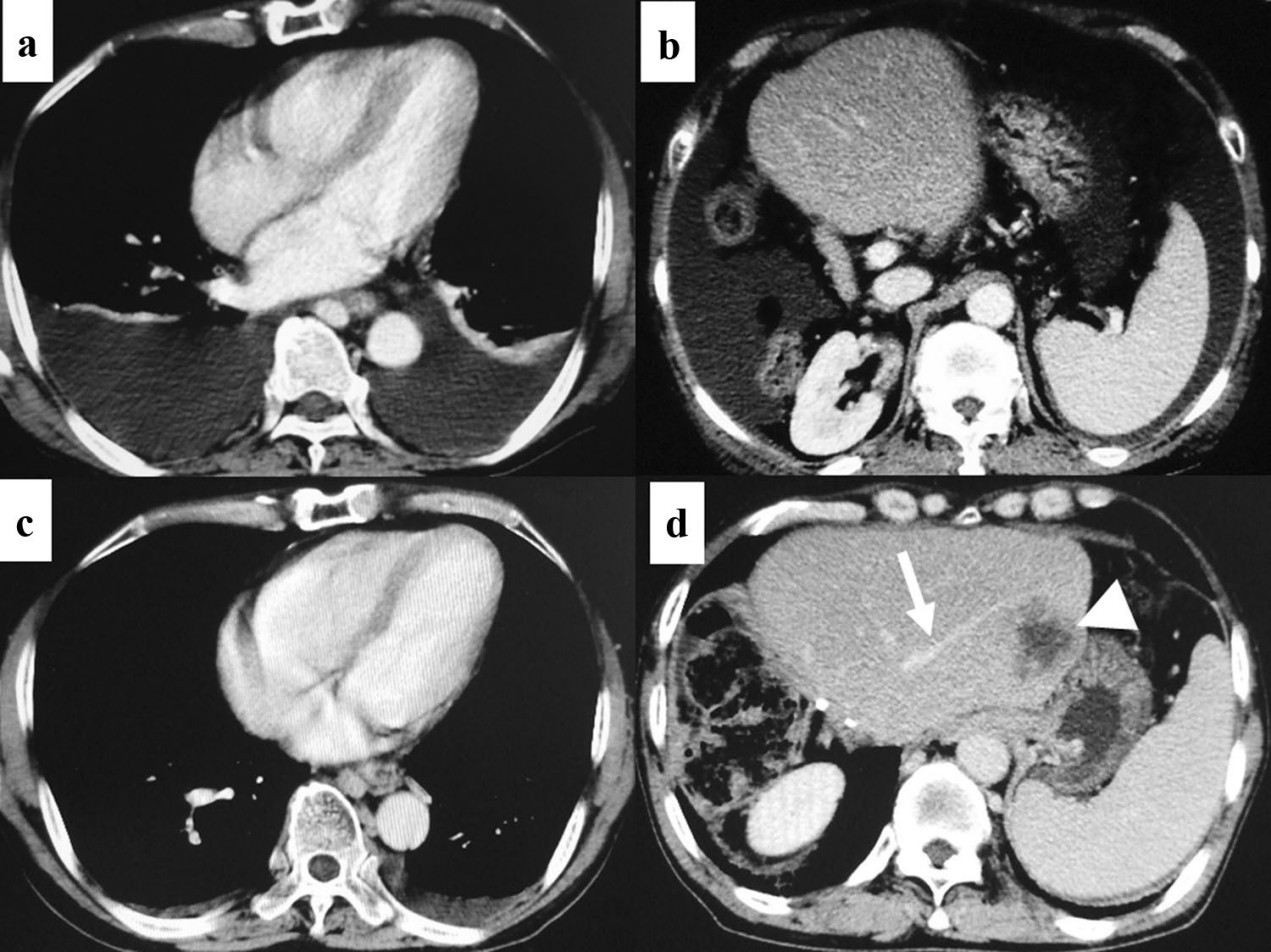
Thoracoabdominal computed tomography findings. Large amount of bilateral pleural effusion (**a**), massive ascites (**b**), and splenomegaly (**b**) were observed in the thoracoabdominal computed tomography taken at the transfer to our institution. Four weeks after initiating the use of tolvaptan, however, pleural effusion (**c**) and ascites (**d**) were almost entirely eradicated. Splenomegaly remained (**d**). A relatively large tumor (white arrowhead, **d**) was observed near the left hepatic vein (white arrow, **d**) in the remaining liver

A 63-year-old man was transferred to our institution for the best supportive care (BSC) for recurrent sigmoid colon cancer. The patient was severely ill at the transfer because the patient had dyspnea due to massive bilateral pleural effusion, massive ascites (Fig. 1a, b), and severe lower body edema.

Two-year previously, the patient received 13 cycles of the OBC (FOLFOX with bevacizumab) followed by the associating liver partition and portal vein ligation for staged hepatectomy (ALPPS) [16] for multiple colorectal liver metastases (CRLM). However, a solitary remaining liver metastasis and multiple (more than 10 lesions) lung metastases recurred 2 months after the ALPPS. Although the patient underwent the endoscopic variceal ligation (EVL) 3 times for esophageal variceal bleeding during the 2-year after the ALPPS, the patient received up to the third-line chemotherapy (the first-line, FOLFOX with bevacizumab; second-line, FOLFIRI with bevacizumab; and third line, irinotecan with cetuximab) until recently. However, shortly after the most recent course of the third-line chemotherapy, the patient suffered from the abovementioned symptoms and was admitted to a tertiary medical center. Because of the marked and sudden deterioration of the patient’s condition which suggested that the patient’s disease entered the terminal stage, the attending doctors recommended the patient to receive the BSC. Despite the diuretics, including furosemide and spironolactone, and albumin infusion, the water-retention symptoms were not improved at all. Thus, the patient accepted the BSC recommendation and thus was transferred to our institution for the BSC.

**Fig. 2:**
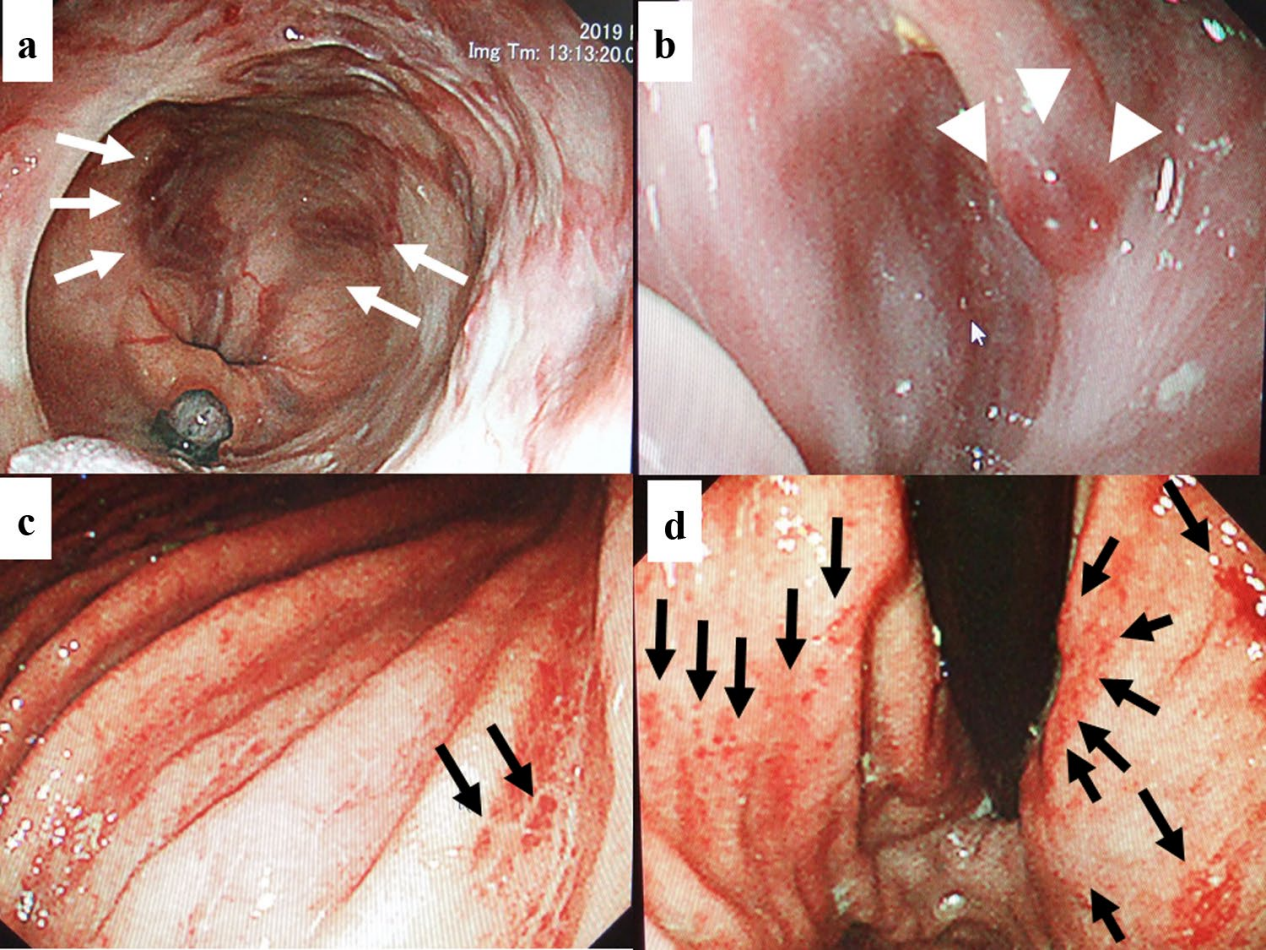
Findings of upper gastrointestinal endoscopy before the shunt surgery. The patient often developed hematemesis and/or melena originating from the esophageal varices and frequently necessitated the endoscopic variceal ligation (EVL). Endoscopic findings that needed the EVL ([red wale markings, white arrows, **a**], [cherry red spot, white arrowheads, **b**]) were frequently observed and the EVL was also performed frequently. Snake-skin-like mosaic pattern of the mucosa and numerous red-brown spots (black arrows) were observed in the lower body (**c**), upper body (**d**), and fundus of the stomach. These findings corresponded to the diagnosis of portal hypertensive gastropathy. Findings of the numerous red-brown spots suggested the severe portal hypertension

**Fig. 3:**
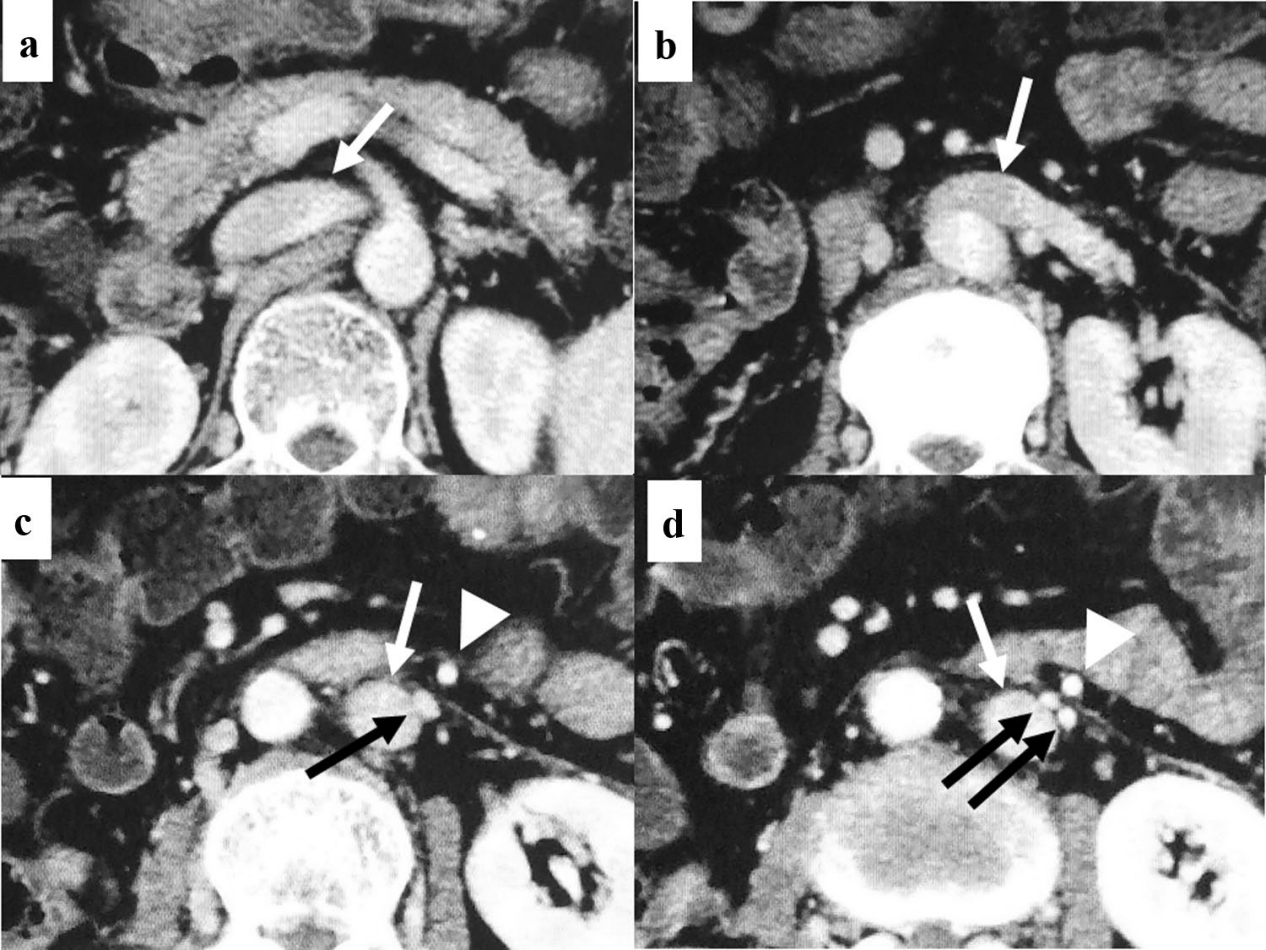
The left-sided inferior vena cava of the present case. The inferior vena cava (IVC) (white arrows) started to shift to the left side at the level of the divergence of the superior mesenteric artery from the abdominal aorta (**a**). As descending to the caudal direction, the IVC passed in front of the aorta (**b**). Below the level of the confluence of the left renal vein and the IVC, the IVC locates in the left side of the abdominal aorta. As shifting to the left side, the IVC (white arrows) gets closer to the inferior mesenteric vein (IMV) (white arrowheads) (**c**, **d**). As further descending to the caudal side, the confluence of the testicular vein trunk and the IVC was observed (**c**, black arrow). At the level of the confluence of the right and left testicular veins (black arrows) (**d**), the IMV (white arrowheads) locates very adjacent to the testicular veins (black arrows)

**Fig. 4:**
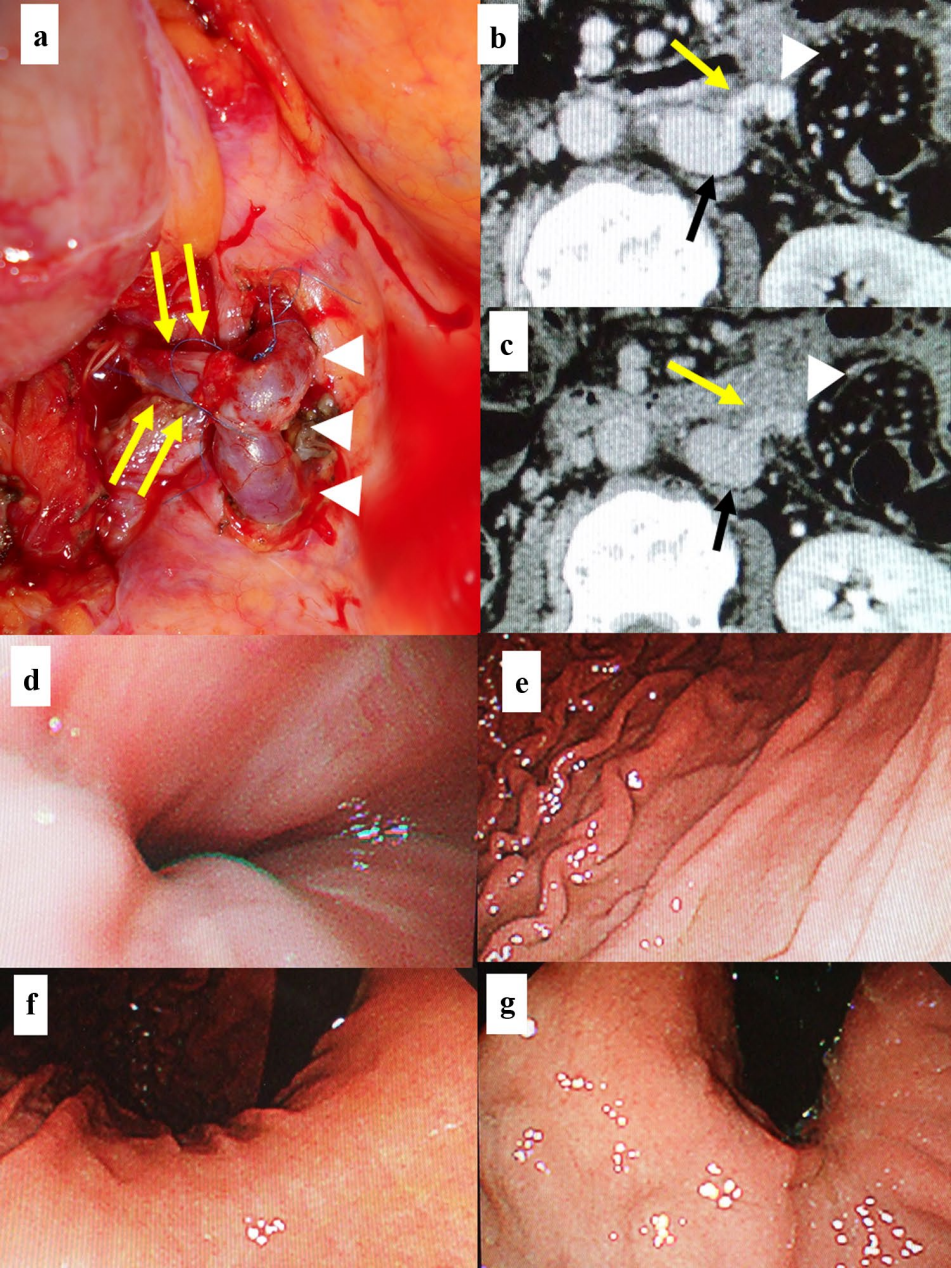
Intraoperative findings of the shunt surgery, abdominal computed tomography findings 2 weeks after the shunt surgery, and upper gastrointestinal endoscopic findings at 2 weeks after the shun surgery. The portosystemic shunt was achieved through the side-to-end-anastomosis of the inferior-mesenteric-vein-to-the-testicular-vein-trunk (**a**). Namely, the cut end of the testicular vein trunk (yellow arrows) was anastomosed to the right lateral wall of the inferior mesenteric vein (IMV) (white arrowheads) (**a**). Portal vein phase of the dynamic contrast-enhancement computed tomography (CECT) (**b**) showed that the IMV (white arrowhead) was connected to the inferior vena cava (IVC) (black arrow) by the testicular vein trunk (yellow arrow). Venous phase of the dynamic CECT (**c**) also demonstrated that the vascular flow from the IMV (white arrowhead) to the IVC (black arrow) was well maintained through the testicular vein (yellow arrow) (**c**). Findings of the upper gastrointestinal endoscopy at 2 weeks after the shunt surgery revealed that findings such as red wale marking, cherry red spot, or hematocystic spot, that need the endoscopic variceal ligation were not observed at all (**d**). Findings of the portal hypertensive gastropathy were nearly eradicated (**e**, **f**, **g**) and the gastric mucosa showed the nearly complete recovery to the normal mucosa (**e**, **f**, **g**)

The patient had the abovementioned treatment history and the episodes of variceal bleeding. In addition, the computed tomography (CT) findings at that time showed the marked splenomegaly (Fig. 1b, d). Moreover, the microscopic findings of the nontumoral background liver, which was sampled at the ALPPS, indicated the SOS (data not shown). Based on these findings, we considered that the patient’s symptoms might have arisen from the portal-hypertension. Therefore, we initiated the use of tolvaptan (7.5 mg a day) [17] immediately after the transfer. After that, the water-retention symptoms were rapidly relieved. Moreover, the patient’s general condition dramatically recovered with the Eastern Cooperative Oncology Group performance status of 0. CT taken 4 weeks after initiating tolvaptan demonstrated the remarkable mitigation of pleural effusion, ascites, and lower body edema (Fig. 1c, d). The patient was discharged from hospital 5 weeks after the transfer. Since then, however, the patient often suffered the esophageal variceal bleeding and frequently underwent the EVL (Fig. 2), which was necessitated 4 times during the 2 months after the discharge. Namely, the EVL could not properly control the variceal bleeding, which disallowed the patient to recommence the antineoplastic treatment. Because hyperammonemia was not observed despite the portal-hypertension, we decided to perform the portosystemic shunt. His recurrent CRLM remained solitary at that time (Fig. 1d) and thus the patient had a possibility of receiving repeat hepatectomy after effective chemotherapy in future. Thus, TIPS was considered a relative contraindication. Hence, we performed surgery consisting of splenectomy and surgical portosystemic shunting 3.5 months after the transfer. Because the patient had a left-sided inferior vena cava (IVC) (Fig. 3a–d), the confluence of the right and left testicular veins existed in the vicinity of the inferior mesenteric vein (Fig. 3c, d). Therefore, the portosystemic shunt was achieved through the side-to-end-anastomosis of the inferior-mesenteric-vein-to-testicular-vein-trunk (Fig. 4a–c) following the splenectomy. Portal venous pressure was altered during the surgery as the following: 25 mmHg before splenectomy, 17 mmHg immediately after splenectomy, and 12 mmHg after the portosystemic shunting. The patient was uneventfully discharged from hospital 7 days after the surgery.

Two-weeks after the surgery, the upper gastrointestinal endoscopy showed that esophageal varices and portal hypertensive gastropathy, both of which were easily detectable before the surgery (Fig. 2), were nearly entirely cured (Fig. 4d–g). Moreover, hyperammonemia did not develop at all throughout the subsequent clinical course. Thereafter, the patient no longer necessitated the EVL. In addition, diuretics, including tolvaptan, could be discontinued entirely: i.e. the shunt surgery was markedly effective. The antineoplastic treatment could be recommenced and continued up to sixth-line until the patient’s death (fourth-line, tegafur-gimeracil-oteracil; fifth-line, regorafenib; sixth-line, trifluridine-tipiracil with bevacizumab).

During the fourth-line chemotherapy, his CRLM remained solitary but remarkably enlarged although his lung metastases continued to show stable disease. Then, the patient strongly hoped to remove the CRLM. Four months after the shunt surgery, we performed repeat hepatectomy (partial hepatectomy) after providing the detailed informed consent that clearly addressed that repeat hepatectomy in such situation was not recommended in various guidelines. The patient was uneventfully discharged from hospital 7 days after the repeat hepatectomy.

Unfortunately, the patient died of deterioration of the multiple lung metastases 1 year after the shunt surgery. Since the discharge from hospital after the shunt surgery, however, the patient did not necessitate any hospitalization other than either the hospitalization for repeat hepatectomy or the hospitalization in which the patient passed away.

## Literature review regarding radiological and/or surgical interventions for the oxaliplatin-based-chemotherapy-associated portal-hypertension

A PubMed search on April 2020 with the key words “oxaliplatin” and “portal hypertension” yielded 27 articles published in English-language journals. These publications were all reviewed. There were only 4 articles describing the 31 cases of radiological and/or surgical interventions for treating the OBC-associated portal-hypertension [7, 9–11]. Interventions included PSE in 25 cases [10], splenic arterial ligation in 4 [9], TIPS in 1 [7], and embolization of the peristomal varices via percutaneous transhepatic portography in 1 [11]. These 31 cases are summarized in Table 1. In this literature review, there were no cases where splenectomy and/or surgical portosystemic shunting was performed.

**Table 1:** A literature review regarding radiological and/or surgical intervention for treating the portal hypertension caused by the oxaliplatin-based chemotherapy

References	Intervention	*n*	Regimen of the OBC*	Number of cycles of the OBC*	Indication of the intervention	Utility of the intervention
Lawal (2012) [7]	TIPS^**†**^	1	FOLFOX with bevacizumab	12	Esophageal variceal bleeding after the failed EVL^‡^	Maintained hemostasis of the variceal bleeding
Schwarz (2014) [9]	SAL^**¶**^ during the hepatectomy surgery	4	Not specified	**≧** 9	Thrombocytopenia High possibility of the post-hepatectomy complications	Increased platelet count Reduced incidence of the post-hepatectomy complications
Luz (2016) [10]	PSE^**∫**^	24 1	FOLFOX FOLFIRINOX	Average 6.4	Discontinuation of the OBC* because of the thrombocytopenia and/or leukocytopenia	Subsequent continuation of the OBC* enabled by the increased leukocyte and platelet counts
Yamaguchi (2018) [11]	Embolization via PTP**	1	FOLFOX with bevacizumab	13	Peristomal variceal bleeding	Obtaining hemostasis of the variceal bleeding otherwise uncontrollable

*Oxaliplatin-based chemotherapy

^**†**^Transjugular intrahepatic portosystemic shunt

^‡^Endoscopic variceal ligation

^**¶**^Splenic arterial ligation

^**∫**^Partial splenic embolization

**Percutaneous transhepatic portography

## Discussion

The hepatotoxicity of the OBC became notorious because the morbidity after hepatectomy for CRLM was more likely to occur in patients who received the OBC before hepatectomy than in those who did not [2, 3, 8, 9, 13–15]. The hepatotoxicity of the OBC is considered attributable to the SOS, i.e. the sinusoidal endothelial injury [2, 13–15]. In other words, the OBC-associated hepatotoxicity originates not directly from the parenchymal injury but indirectly from the sinusoid-circulatory disturbance caused by the SOS [2, 15]. In early 2000s, the OBC-associated hepatotoxicity was considered problematic only when performing hepatectomy [1–3, 5, 6, 8, 9]. However, the portal-hypertension has been reported as the significant clinical manifestation of the SOS from the late 2000s [4–7, 10–15]. Thereafter, the treatment of the OBC-associated portal-hypertension itself has been often reported [4, 7, 9–14].

In this case, the water-retention symptoms of the portal-hypertension, such as pleural effusion, ascites, or edema, were mitigated by the use of tolvaptan [17]. However, the variceal bleeding increased in frequency and became unable to be managed with the EVL despite the markedly improved patient’s condition. Thus, we performed the shunt surgery. The purpose of this surgery was not only to treat the variceal bleeding but also to recommence the antineoplastic treatment. Thus, we performed splenectomy simultaneously with the surgical portosystemic shunt. Namely, splenectomy can bring the mitigation of the portal-hypertension as well as the prevention of pancytopenia caused by the antineoplastic treatment [10]. Combined TIPS [7] and PSE [10] may be able to bring the effect similar to the effect brought on by combined splenectomy and surgical portosystemic shunting. However, the liver of the patient was the remaining liver after receiving the ALPPS (extended right hemi-hepatectomy). Namely, the patient’s left hepatic vein, which would be used for stenting if performing TIPS in this case, was the sole major hepatic vein. Thus, performing the TIPS for this case leads to the abandonment of receiving future repeat hepatectomy. Therefore, we performed the surgical portosystemic shunting. The fact that the patient was able to undergo repeat hepatectomy supports the reasonability of our choice although the disease cure was not brought on by the repeat hepatectomy.

As abovementioned, the hepatotoxicity of the OBC is not caused by the hepatocyte injury itself but caused by the SOS [15]. Therefore, hyperammonemia rarely occurs in the OBC-associated portal-hypertension [3–15]. Thus, the portosystemic shunt is considered reasonable for the treatment of the OBC-associated portal-hypertension. In this case, the inferior-mesenteric-vein-to-testicular-vein-trunk-shunt was easily performed because of the adjacency of these veins due to the left-sided IVC (Fig. 4a–c). Even in cases of the normal right-sided IVC, however, the left testicular vein locates in vicinity of the inferior mesenteric vein. Thus, the portosystemic shunt through the inferior-mesenteric-vein-to-left-testicular-vein may be a promising choice for surgical portosystemic shunting.

## Abbreviations

OBC: Oxaliplatin-based chemotherapy
SOS: Sinusoidal obstruction syndrome
PSE: Partial splenic embolization
TIPS: Transjugular intrahepatic portosystemic shunt
BSC: Best supportive care
ALPPS: Associating liver partition and portal vein ligation for staged hepatectomy
CRLM: Colorectal liver metastasis
EVL: Endoscopic variceal ligation
CT: Computed tomography
IVC: Inferior vena cava
